# Supplementary material for: Dietary restriction of cysteine and methionine sensitizes gliomas to ferroptosis and induces alterations in energetic metabolism
Source: Nat Commun. 2023 Mar 2;14:1187. doi: 10.1038/s41467-023-36630-w (PMC9981683; doi:10.1038/s41467-023-36630-w)
Supplement: Supplementary file 1 — Supplementary Figures and Tables [file 41467_2023_36630_MOESM1_ESM.pdf]

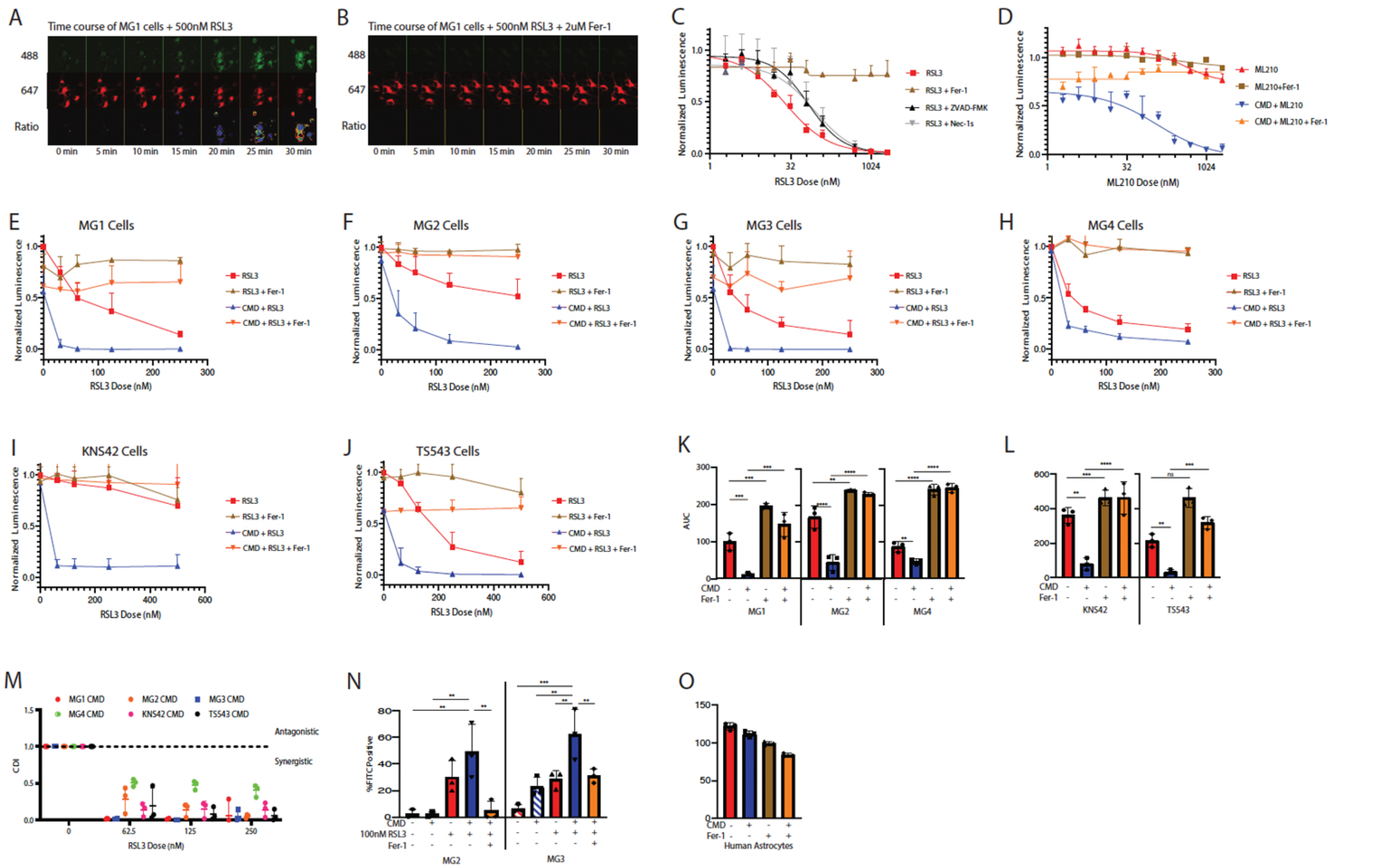

Supplementary Figure 1. *In vitro effects of CMD* (A) Live cell confocal microscopy of Bodipy-C11 labeled MG1 cells treated with 500nM RSL3, added at time 0 minutes. (B) Live cell confocal microscopy of Bodipy-C11 labeled MG1 cells with 500nM RSL3 and 2µM Ferrostatin-1 added at time 0 minutes. Upper panels show the oxidized, middle panels the reduced, and bottom panels the ratio of oxidized/reduced Bodipy-C11. Each frame = 100 µm ´ 100 µm. (C) Representative dose-response of MG1 cells treated with RSL3 (red), RSL3 plus Ferrostatin-1 (brown), RSL3 plus 5uM ZVAD-FMK (black), RSL3 plus 2µM Nec-1s (gray). (D) Representative dose-response showing MG3 cell response to ML-210 (red), ML-210 plus 2uM Ferrostatin-1 (brown), CMD + ML-210 (blue), CMD + ML-210 + 2µM Ferrostatin-1 (orange). (A)-(D) experiments were run 3 independent times with similar results and representative figures shown. (E)-(J) Dose-response curves for MG1(E), MG2 (F), MG3 (G), MG4 (H), KNS42 (I), TS543 (J) glioma cells treated with RSL3 ± CMD ± 2µM Ferrostatin-1. Each data point is an average of 3 independent experiments with each dose having 3 technical replicates. (K) AUC quantification for dose-response curves from three murine glioma cell lines treated with RSL3 ± CMD ± 2uM Ferrostatin-1. (L) AUC quantification for dose response curves from two human glioma cell lines treated with RSL3 ± CMD ± 2µM Ferrostatin-1. (K)-(L) AUC averaged across 3 independent experiments. (M) Chou-Talaly quantification of the coefficient of drug interaction quantification for the cell viability data from all murine and human glioma cell lines treated with RSL3 and CMD for 24 hours. (N) Quantification of 3 independent flow cytometry experiments using Bodipy-C11 for two murine glioma cell lines (MG2, MG3). (O) AUC quantification for dose response curves for human astrocyte cell lines treated with RSL3 ± CMD ± 2µM Ferrostatin-1 with data averaged across 3 independent experiments. All bar graphs presented as mean ± SD. Significance denoted by: Significance denoted by: * - p<0.05, ** -p<0.01, *** - p<0.001.

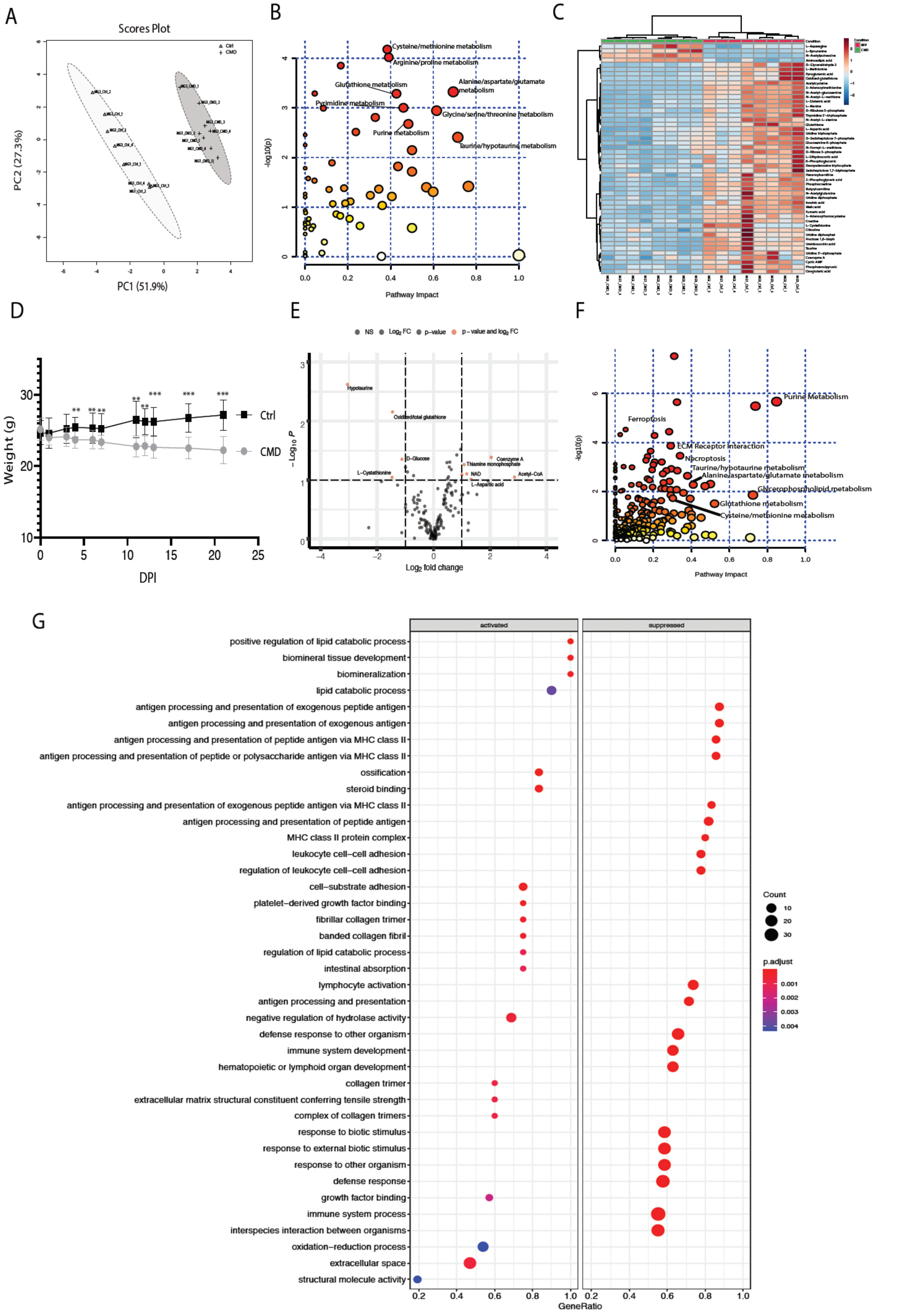

Supplementary Figure 2. *(A)-(C) In vitro metabolite profiling data from MG3 cell lines.* (A) Principal component analysis of metabolite profiling showing clustering along treatment conditions (light grey=control, dark grey=CMD). (B) Pathway analysis of targeted metabolite profiling across control and CMD samples spanning 200 metabolites with relative concentrations log transformed and samples scaled by mean. Labeled pathways have FDR<.05. (C) Heatmap showing top 50 differentially assessed metabolites based on FDR-corrected p-value, all <0.05. *(D)-(G) in vivo metabolomic/proteomic data from end stage MG3 tumors.* (D) Weights from C57/B6 male mice put on control (n=8) or CMD diet (n=8) with data presented as mean ± SD. Two-tailed t-tests performed to assess significance. **p<0.01, *** p<0.001 (E) Volcano plot with labeled metabolites having p<0.1 and LFC>|1| from targeted metabolite profiling comparing control (n=4) and CMD (n=5) male mice with relative concentrations log transformed and samples scaled by mean. (F) Joint pathway analysis combining proteomics data of differential expression analysis comparing CMD vs. control (FDR <0.2, |LFC|>0.58) and metabolite differential assessment analysis (|LFC|>0.58) comparing CMD vs. control. Enrichment analysis using hypergeometric test and integration method based on queries. Relevant pathways with FDR <0.1 labeled. (G) Dot plot of top 20 suppressed/activated protein/gene sets based on untargeted protein level enrichment analysis of FFPE end-stage samples from control (n=3) and CMD (n=4) male mice. Significance denoted by: Significance denoted by: * - p<0.05, ** -p<0.01, *** - p<0.001.

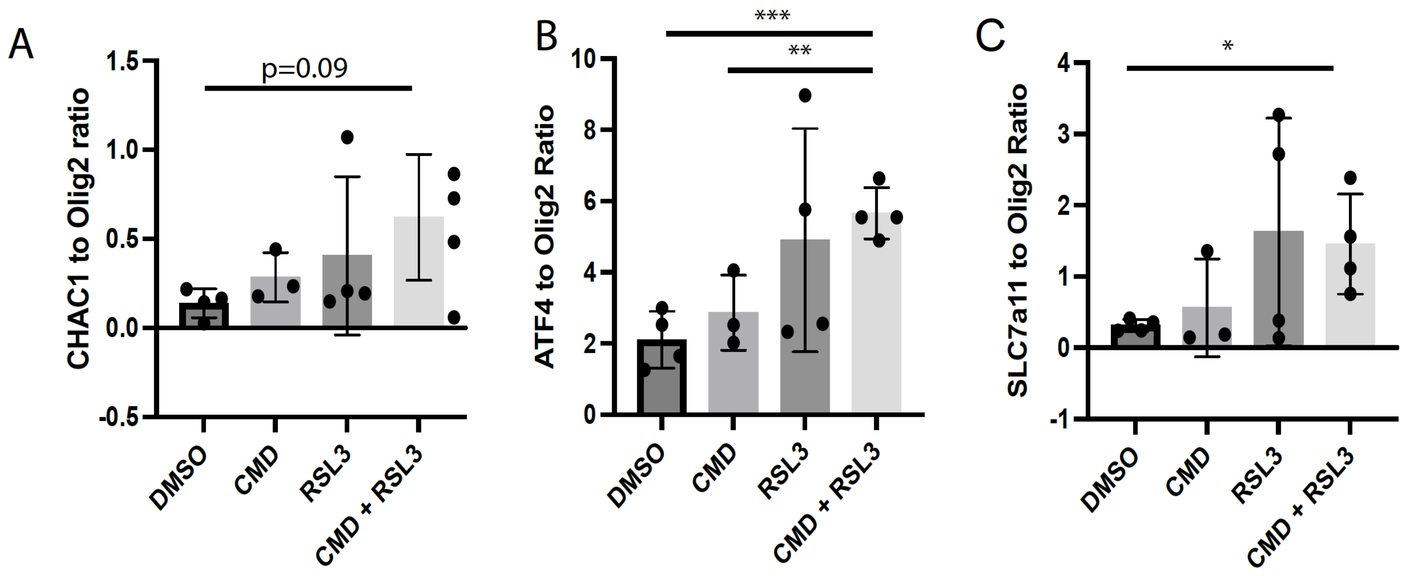

Supplementary Figure 3. RT-qPCR validation of ferroptosis markers after acute *in vivo* treatment. RT-qPCR data for (A) CHAC1 (B) ATF4 (C) SLC7a11 transcripts. C57/B6 female mice were injected with MG3 cells and, at 28 days post injection, were treated for 3 days with DMSO, CMD diet, RSL3 or CMD diet plus RSL3. Tissue was collected immediately after treatment. To account for depletion of tumor cells within treatment groups, transcript expression was normalized to expression level of Olig2, a tumor marker. Data plotted as expression level for gene of interest relative to Actin (2-ΔCt) over expression level of Olig2 relative to Actin with bar graphs presented as mean ± SD. Statistics assessed using two-tailed t-tests on paired ratios (control n=4, CMD n=3, RSL3 n=3, CMD+RSL3 n=4). Significance denoted by: Significance denoted by: * - p<0.05, ** -p<0.01, *** - p<0.001.

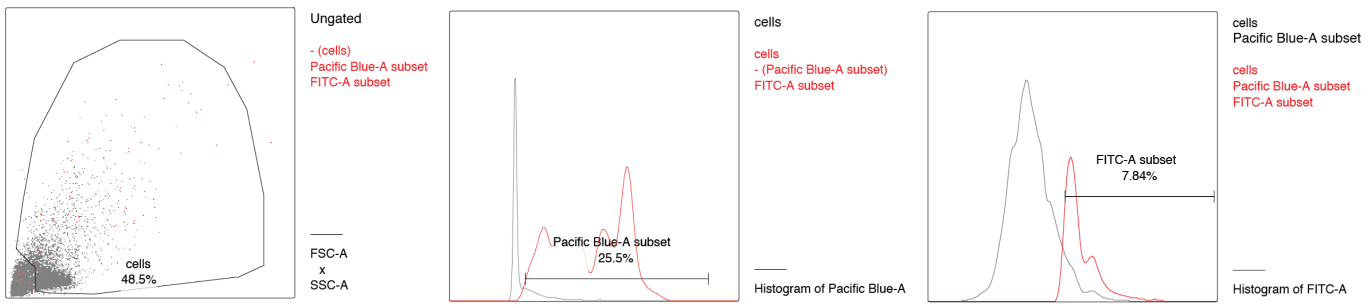

Supplementary Figure 4. Slice culture flow cytometry back-gating. Example of dissociated slice culture sample with dissociated cells run through a flow cytometer after staining with Calcein Blue and H2DCFDA. Samples were gated off FSC x SSC to determine cell population separate from debris. Next, live cells were selected using a Calcein-Blue stain gated based off a negative control (unstained sample without Calcein-Blue). The live cells (Calcein-Blue) were then gated off FITC-H2DCFDA to determine live cell ROS.

Supplementary Table 1				
Lab Designation	***Nomenclature in Paper***	***Species***	***Genetic Background***	***Details***
333 [36]	mouse-glioma-1 (MG1)	Mouse	p53-/-, PDGFA overexpressing	Diffusely infiltrating phenotype
ACre	MG2	Mouse	p53-/-, PDGFA overexpressing	Diffusely infiltrating phenotype
APCL	MG3	Mouse	p53-/-, PDGFA overexpressing	Diffusely infiltrating phenotype
MGPP3 [35]	MG4	Mouse	p53-/-, PTEN -/-, PDGFB overexpressing	Aggressive, psuedopalisading necrosis
TS543	TS543	Human	Human GBM Culture - PDGFR-A amplified	Proneural
KNS42	KNS42	Human	Pediatric GBM Culture - p53 mutated, H3 G34R mutant	Mesenchymal

Supplementary Table 1. Cell line designation used within manuscript.

Lipid Species	FC - Ctrl/CMD	log2FC	p-Value
**FA 16:0**	0.616702938	-0.697352375	0.042978423
**FA 16:1**	0.752902502	-0.409465041	0.553822858
**FA 17:0**	0.635974987	-0.652958069	0.147440016
**FA 17:1**	0.691940797	-0.53127949	0.335168664
**FA 18:2**	0.495326418	-1.013548527	0.33300642
**FA 18:3**	0.596288558	-0.74591744	0.54131885
**FA 20:1**	0.698205843	-0.518275664	0.15552286
**FA 20:5**	0.997956403	-0.002951304	0.996543825
**FA 22:4**	0.912979647	-0.131345397	0.703434415
**PE 40:6**	1.512321394	0.596764769	0.092217297
**PE 44:11**	1.173066336	0.230284599	0.039779938
**PE O-36:2**	1.645000275	0.718087826	0.091058961
**PE O-38:7**	1.53124605	0.614706123	0.064309482
**PE O-40:7**	1.646103479	0.719055031	0.053036419
**PG 32:0**	0.72319399	-0.467545405	0.432703343
**PG 36:1**	1.374852389	0.459276732	0.091114536
**PG 36:4**	0.702376201	-0.509684133	0.234694845
**PG 38:4**	0.606565267	-0.721265205	0.122647498
**PI 38:5**	1.077540565	0.107742181	0.61452003
**PI 38:6**	1.072088293	0.100423726	0.303821259
**PC O-38:7**	1.428509971	0.514511106	0.072306948
**PS 40:6**	0.944546797	-0.08230582	0.816263285
**PS 40:7**	1.251022582	0.323107831	0.25411951
**PS 44:10**	1.158683651	0.212486729	0.064644309

Supplementary Table 2. DESI Lipidomic analysis of Non-Tumor Area; Ctrl (n=4) versus CMD (n=4). Fold change is calculated for the average signal intensity of control versus CMD. And, p value is calculated from t-test.

Supplementary Table 3. List of oligonucleotides sequences for qPCR studies	
**Primer Transcript Name**	**Oligo Sequence (5'-3')**
Human beta-Actin Forward	CATGTACGTTGCTATCCAGGC,
Human beta-Actin Reverse	CTCCTTAATGTCACGCACGAT,
Human SLC7a11 Forward	TCTCCAAAGGAGGTTACCTGC
Human SLC7a11 Reverse	AGACTCCCCTCAGTAAAGTGAC
Human ATF4 Forward	ATGACCGAAATGAGCTTCCTG
Human ATF4 Reverse	GCTGGAGAACCCATGAGGT
Mouse beta-Actin Forward	CGAGGCCCAGAGCAAGAGAG
Mouse beta-Actin Reverse	CTCGTAGATGGGCACAGTGTG
Mouse ATF4 Forward	CCTGAACAGCGAAGTGTTGG
Mouse ATF4 Reverse	TGGAGAACCCATGAGGTTTCAA
Mouse SLC7a11 Forward	GGCACCGTCATCGGATCAG
Mouse SLC7a11 Reverse	CTCCACAGGCAGACCAGAAAA
Mouse PTGS2 Forward	TTCAACACACTCTATCACTGGC
Mouse PTGS2 Reverse	AGAAGCGTTTGCGGTACTCAT
Mouse/Human Chac1 Forward	CTGTGGATTTTCGGGTACGG
Mouse/Humans Chac1 Reverse	CCCTATGGAAGGTGTCTCC

Supplementary Table 3. List of qPCR primers
